# Unraveling the Challenges of Difficult-to-Treat Spondyloarthritis: SPARTAN 2024 Annual Meeting Proceedings

**DOI:** 10.1007/s11926-025-01183-y

**Published:** 2025-02-03

**Authors:** Andre L Ribeiro, Fabian Proft

**Affiliations:** 1 Division of Rheumatology, Hospital de Clínicas de Porto Alegre, Porto Alegre, Brazil; 2 Division of Rheumatology, Hospital Moinhos de Vento, Porto Alegre, Brazil; 3 Department of Gastroenterology, Infectiology and Rheumatology (including Nutrition Medicine), Charité – Universitätsmedizin Berlin, corporate member of Freie Universität Berlin and Humboldt-Universität zu Berlin, Hindenburgdamm 30, 12203 Berlin, Germany

**Keywords:** Spondyloarthritis - therapy, BDMARDs, CsDMARDs - treatment failure, Difficult to treat

## Abstract

**Purpose of Review:**

This review aims to explore the emerging concept of difficult-to-treat axial spondyloarthritis (D2T-axSpA), including its definitions, clinical challenges, and management strategies. The objective, as presented at the SPARTAN 2024 Annual Meeting, is to delineate the evolving framework for identifying and addressing D2T-axSpA, with a focus on inflammatory and non-inflammatory mechanisms of treatment failure and the implications for clinical practice.

**Recent Findings:**

Studies have highlighted a prevalence of D2T-axSpA ranging from 19.5 to 28.3% in real-world cohorts, with associated risk factors including peripheral arthritis, comorbidities, and female gender. Recent advances include the Assessment of SpondyloArthritis International Society’s (ASAS) preliminary definition of “difficult-to-manage axSpA” (D2M-axSpA), which encompasses treatment-refractory cases and broader management challenges and `treatment refractory axSpA´ where objective evidence of ongoing inflammation is mandatory.

**Summary:**

D2T-axSpA presents significant challenges due to persistent disease activity and the interplay of inflammatory and non-inflammatory drivers. The emerging definitions and research into personalized treatment strategies promise to refine clinical management. Future directions emphasize biomarker-driven precision medicine, novel therapeutic combinations, and holistic care models to improve outcomes in this complex patient population.

## Introduction

Spondyloarthritis (SpA) encompasses a group of chronic inflammatory diseases with shared clinical, genetic, and pathophysiological features, including axial skeleton involvement, peripheral manifestations, and extra-articular conditions such as uveitis, psoriasis, and inflammatory bowel disease (IBD) [1]. Advances in classification, particularly by the Assessment of SpondyloArthritis International Society (ASAS) criteria, have provided a more inclusive framework for diagnosing axial spondyloarthritis (axSpA), incorporating both radiographic and non-radiographic forms [2]. This has enabled earlier recognition of non-radiographic cases that were previously underdiagnosed by the modified New York criteria [3], but had already been described in the literature [4]. The two most prevalent conditions within the spectrum of SpA are axial spondyloarthritis (axSpA), encompassing both radiographic (previously known as ankylosing spondylitis – AS) and non-radiographic forms, and psoriatic arthritis (PsA).

In the management of axSpA, the ASAS/EULAR recommendations emphasize a treat-to-target approach, where the target – decided through shared decision-making between patient and rheumatologist – is aimed at achieving sustained clinical remission or low disease activity [5]. These guidelines underscore the importance of early and continuous treatment adjustments to control symptoms and prevent long-term damage. Achieving clinical responses, such as the ASAS40 response or according to the Axial Spondyloarthritis Disease Activity Score (ASDAS), leads to significant improvements in physical function, quality of life, and work productivity in patients with axSpA [6]. However, only approximately 40–50% of patients reach ASAS40 with current therapies [7], and a mere 17.6% sustain ASDAS remission [8].

Despite the approval of several medications incorporated into the latest treatment guidelines [5], a significant proportion of patients still fail to achieve adequate treatment response. This raises important questions about the underlying reasons for these failures and the concept of difficult-to-treat (D2T) axSpA (D2T-axSpA). As highlighted in a recent editorial by our group, D2T-axSpA is an emerging challenge that requires refined definitions and personalized management strategies to address persistent disease activity and treatment failures [9]. Underscoring the urgency of this need, recent studies that extrapolated D2T criteria from rheumatoid arthritis (RA) to axSpA have reported the prevalence of D2T-axSpA ranging from 19.5 to 28.3% in real-world cohorts [10, 11].

### Understanding the Clinical Challenges in Difficult-to-Treat Axial Spondyloarthritis

Due to the high prevalence and associated morbidity, D2T-axSpA received significant attention at the SPARTAN 2024 Annual Meeting. In RA, the concept of D2T is more clearly defined: patients are classified as D2T after failing one conventional synthetic disease-modifying antirheumatic drug (csDMARD) and at least two biological or target synthetic DMARDs (b/tsDMARDs) with different mechanisms of action. Additionally, the disease must remain objectively active and considered problematic by either the patient or the rheumatologist [12].

In contrast, the definition of D2T in axSpA is still evolving, reflecting its complexity and variability in disease presentation. However, significant progress was recently made by the ASAS group. In January 2024, ASAS endorsed a preliminary definition of difficult-to-manage (D2M) axSpA (D2M-axSpA), a broader concept that captures not only treatment failure, but also suboptimal disease control and the recognition of problematic signs and symptoms by patients and/or physicians [13].

The ASAS D2M-axSpA definition includes several criteria [13]: [1] failure of at least two b/tsDMARDs with different mechanisms of action (or contraindications) [2], insufficient control of signs/symptoms, such as high disease activity (ASDAS ≥ 2.1), objective signs of inflammation (elevated C-reactive protein [CRP] or MRI-detected inflammation), or rapid radiographic progression, and [3] disease that remains problematic for the patients and/or physician despite otherwise controlled metrics. This structured definition also provides a clear framework for identifying treatment-refractory axSpA, a subset of D2M-axSpA characterized by ongoing inflammation despite standard treatments (“treatment refractory axSpA”). In these cases, in addition to high disease activity, the presence of objective signs of ongoing inflammation is mandatory (i.e., elevated CRP or inflammation visible on MRI).

This distinction between inflammatory and non-inflammatory mechanisms is essential to understanding treatment failure in axSpA and is central to the ASAS definition. Persistent inflammation in treatment-refractory axSpA may necessitate switching therapies or exploring combination therapy approaches, while non-inflammatory drivers – such as chronic pain and depression – require alternative management strategies. Therefore, identifying the underlying drivers of D2M-axSpA, whether due to persistent inflammation or factors such as comorbid conditions and patient-related issues, is crucial for improving outcomes. McGonagle et al. emphasize the need to distinguish between persistent inflammatory refractory axSpA (PIRaxSpA), where ongoing inflammation aligns with ASAS’s treatment-refractory criteria, and non-inflammatory refractory axSpA (NIRaxSpA), which aligns with the broader D2M definition and involves symptoms driven by factors like chronic pain, central sensitization, or fibromyalgia rather than persistent inflammation [14].

The challenges in defining and managing D2T-axSpA parallel those seen in PsA. Similar to axSpA, PsA is a complex disease that affects multiple domains, including peripheral joints, skin, and entheses [15]. The Group for Research and Assessment of Psoriasis and Psoriatic Arthritis (GRAPPA) has taken steps to define D2T- and complex-to-manage PsA (D2T-PsA and C2M-PsA). The classification of D2T-PsA focuses on persistent inflammation despite multiple therapies, akin to PIRaxSpA, while C2M-PsA also involves treatment failures due to non-inflammatory factors, much like NIRaxSpA [16, 17].

In both PsA and axSpA, a careful reassessment of the diagnosis is critical before applying these definitions and escalating therapy. Once the patients meet the criteria for D2T, an interdisciplinary management approach—encompassing pharmacological treatments, physical therapy, mental health support, and lifestyle modifications—is essential to optimize outcomes [18].

### The Characteristic of Difficult-to-Treat Axial Spondyloarthritis Patients

Currently, there is limited information available on the characteristics of D2T-axSpA patients, and much of the existing knowledge is derived from extrapolations of the D2T-RA definition to real-world cohorts. These studies have been instrumental in offering preliminary insights, but they highlight the need for further research to develop a robust, axSpA-specific framework.

One of the recently published cohorts on D2T-axSpA was conducted by Philippoteaux et al., a multicenter retrospective cohort study based in Northern France across three centers, covering the period from January 2018 to January 2021 [11]. The study included 311 patients aged 18 and older, diagnosed with axSpA according to ASAS criteria, without requiring imaging confirmation. D2T-axSpA was defined as the failure of at least two b/tsDMARDs with different mechanisms of action, or failure of one b/tsDMARD if there was a contraindication to a second therapeutic class (e.g., TNF inhibitors or IL-17 inhibitors). The study also classified a subset of patients as very difficult-to-treat (vD2T), defined by meeting the D2T definition within two years of initiating therapy. Among the cohort, 88 patients (28.3%) met the criteria for D2T-axSpA, and 12 (3.8%) were classified as vD2T.

In terms of clinical characteristics, the study identified several key differences between D2T-axSpA and non-D2T (nD2T) patients in the univariate analysis. Peripheral arthritis was more common in the D2T group (34.9% vs. 21.4%), and disease activity, as measured by BASDAI scores, was higher in the D2T group (63.7 vs. 58.8). Uveitis and fibromyalgia were also more prevalent among D2T patients, at 21.2% and 17.4% respectively, compared to 11% and 4% in the nD2T group. However, the multivariate analysis did not reveal any clear predictive factors for D2T-axSpA, suggesting that multiple overlapping factors may contribute to treatment resistance. Among vD2T patients, IBD, uveitis, and elevated CRP levels were more common. Despite these findings, the study had several limitations, including the potential underestimation of fibromyalgia and comorbidities due to inconsistent documentation in the electronic medical records. The small sample size, particularly for vD2T patients, also limited the statistical power of the analysis.

Fakih et al. conducted a large-scale nationwide observational study using the French National Medico-Administrative Database (SNDS) to explore the prevalence and characteristics of D2T-axSpA [10]. The retrospective cohort, which included data from January 2010 to December 2018, enrolled 22,932 patients with AS based on the ICD code M45, without requiring imaging evidence. D2T-axSpA was defined as failure of at least three b/tsDMARDs in total, or failure of at least two b/tsDMARDs with different mechanisms of action. Among the 10,798 patients exposed to at least one bDMARD, 2,115 (19.59%) met the criteria for D2T-axSpA.

Key findings from the study revealed that D2T-axSpA was more common among women (66.5% vs. 50.7%) and in patients with peripheral arthritis, psoriasis, obesity, smoking, hypertension, and depression. No significant differences were observed between D2T and nD2T patients in terms of uveitis or IBD. In the multivariate analysis, female gender, peripheral arthritis, psoriasis, hypertension, and depression remained significantly associated with D2T-axSpA. The study also noted limitations, including restricting the analysis to patients exposed to bDMARDs, which may not reflect the broader axSpA population. Additionally, coding biases may have influenced the findings, as M45 was at the time only used for AS, potentially underrepresenting non-radiographic axSpA cases. Moreover, including patients from 2010, when fewer biologics were available, may have affected the study’s outcomes. Despite these limitations, the study concluded that D2T-axSpA affects approximately one in five patients exposed to bDMARDs and is strongly associated with comorbid conditions, even though it did not analyze for concurrent fibromyalgia.

While these studies provide valuable insights into the characteristics of patients with D2T-axSpA, they also underline the current reliance on RA-based definitions and the need for axSpA-specific criteria. Key factors associated with D2T-axSpA included higher disease activity scores at baseline, peripheral arthritis, and comorbid conditions such as hypertension and depression. There was conflicting data regarding obesity, uveitis, and IBD. More research will be critical in refining the definition of D2T-axSpA and determining predictors for its development.

### Future Directions and Ongoing Research

Looking ahead, as discussed at the SPARTAN 2024 Annual Meeting, the future directions in managing D2T-axSpA are closely tied to the development of a specific definition for axSpA and new ways to better identify and treat these patients. Research into biomarkers that can predict treatment response will be vital in tailoring treatments to individual patients towards A “*personalized medicine*” approach, allowing clinicians to identify patients at higher risk of becoming D2T before they begin therapy, thus enabling proactive adjustments to their treatment.

Furthermore, as rheumatologists seek to refine treatment strategies, the role of combined b/tsDMARD therapies is gaining increasing attention (i.e., simultaneous use of bDMARDs or a combination of a bDMARD with a tsDMARD). While current guidelines recommend a stepwise approach using single-agent b/tsDMARDs, emerging research suggests that combining therapies with different mechanisms of action may provide more comprehensive control of inflammation, with small to no increase in the risk of infectious adverse events [19–21]. For example, combining an IL-17 or IL-23 inhibitor with a JAK inhibitor may be beneficial for certain patients with treatment-refractory axSpA. Supporting this concept, the VEGA trial demonstrated that the combination of guselkumab (an IL-23 inhibitor) and golimumab (a TNF-alpha inhibitor), when compared to golimumab alone, increased efficacy without a rise in infections or other side effects in patients with ulcerative colitis [20]. Building on these findings, the ongoing AFFINITY study (NCT05071664) is evaluating a similar combination approach in patients with PsA [22]. These studies highlight the potential benefits of combining biologic therapies for patients who have had an inadequate response to previous treatments, potentially offering future strategies for managing complex cases.

**Fig. 1:**
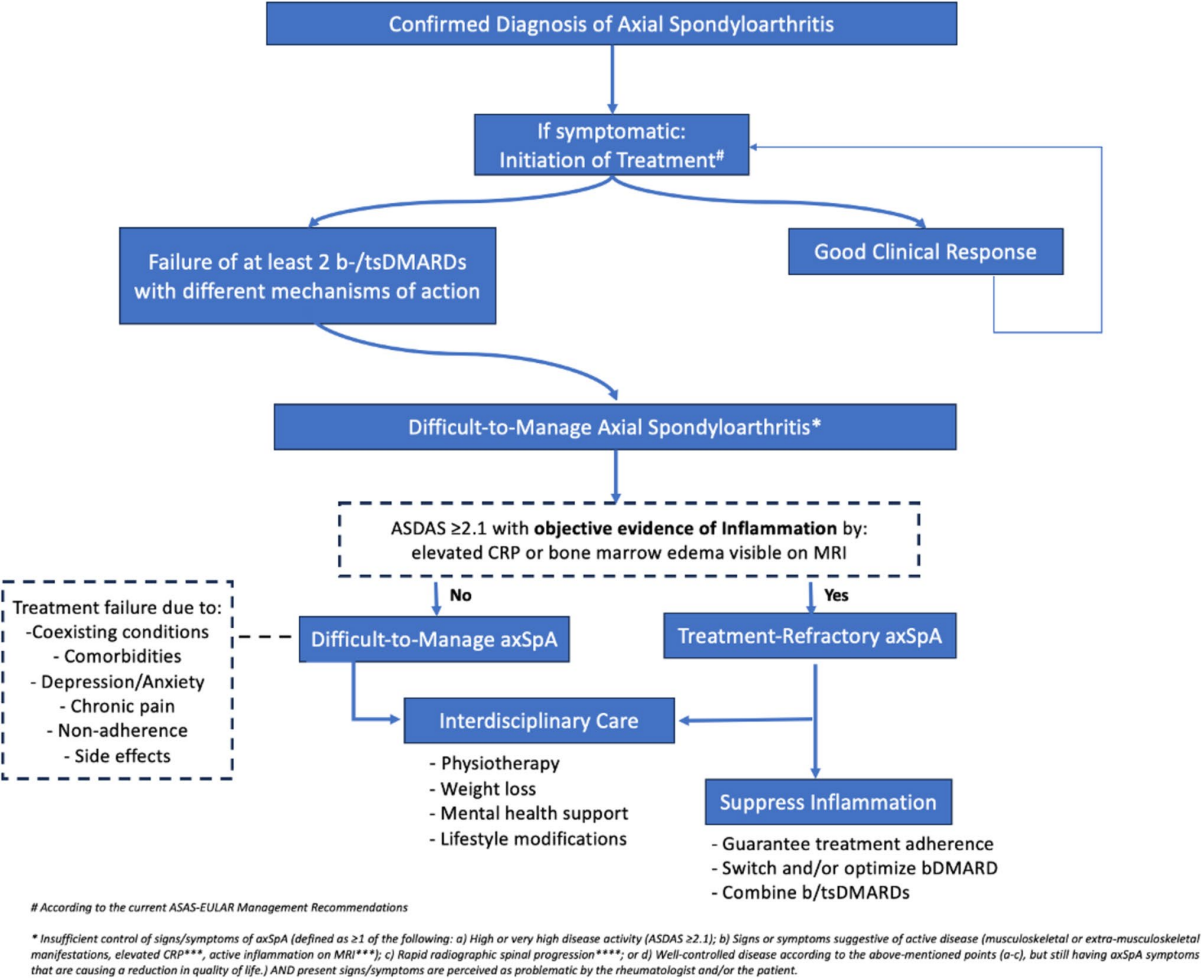
Understanding Difficult-to-Manage Axial Spondyloarthritis (axSpA)

As discussed, the ASAS has already endorsed a definition for D2M-axSpA and treatment-refractory axSpA, and the associated publication is underway, aiming to provide a much-needed standardized framework for the identification and management of these challenging cases. This concept, which parallels the D2T approach in RA, incorporates criteria tailored to the unique clinical features of axSpA. By integrating both objective clinical and imaging data with patient-reported outcomes, the definition captures the complex nature of D2M-axSpA, addressing both inflammatory and non-inflammatory factors, such as central sensitization and comorbidities. Once published, this framework will help clinicians identify D2T-axSpA cases more effectively, enabling more targeted treatment strategies and minimizing unnecessary therapeutic escalations, ultimately improving patient outcomes (Fig. 1).

This figure outlines the spectrum of D2M-axSpA, emphasizing the distinction between general management challenges and the subset of treatment-refractory axSpA. The broader category of D2M-axSpA encompasses all patients with unsatisfactory treatment outcomes, stemming from a variety of factors such as comorbidities, chronic pain, or non-inflammatory drivers. In contrast, treatment-refractory axSpA represents a smaller subset, defined by active disease (ASDAS ≥ 2.1) with objective signs of ongoing inflammation, such as elevated CRP or bone marrow edema on MRI. Management strategies vary based on the subset and may include targeting inflammation, addressing comorbidities, optimizing therapies, and incorporating interdisciplinary care approaches.

## Conclusion

In conclusion, D2T-axSpA presents significant challenges for both patients and clinicians. The evolving definition of D2T-axSpA will be instrumental in guiding treatment decisions, improving outcomes for this complex patient population and stimulate future research projects on this important topic. Drawing parallels with PsA offers valuable insights into how interdisciplinary care and personalized treatment strategies can benefit axSpA patients, particularly when addressing both inflammatory and non-inflammatory drivers of the disease.

As the field continues to evolve, future research into biomarkers, dual therapies, and personalized medicine approaches will be key to refining treatment algorithms for D2T-axSpA. These approaches promise enhanced disease control through the strategic combination of biologics and also highlight the importance of pre-emptive treatment adjustments based on predictive biomarkers. By adopting a holistic, patient-centered approach that addresses not only disease activity but also the broader context of patient well-being, rheumatologists can better unravel the challenges of managing D2T-axSpA.

## Key References

- **Sieper J**,** Poddubnyy D. Axial spondyloarthritis. Lancet. 2017 Jul 1;390(10089):73–84.**
  This foundational review provides an in-depth understanding of axSpA, its clinical features, and its management challenges.
- **Philippoteaux C**,** Delepine T**,** Cailliau E**,** et al. Characteristics of difficult-to-treat axial spondyloarthritis: Results of a real-world multicentric study. Joint Bone Spine. 2024 Mar;91** [2]:**105,670.**
  This multicenter study provides insights into the prevalence and characteristics of D2T-axSpA, reporting that 28.3% of axSpA patients meet the criteria for D2T-axSpA. The study also identified key risk factors, including peripheral arthritis and comorbidities.
- **Fakih O**,** Desmarets M**,** Martin B**,** et al. Difficult-to-treat axial spondyloarthritis is associated with psoriasis**,** peripheral involvement**,** and comorbidities: Results of an observational nationwide study. RMD Open. 2023 Nov 23;9** [4]:**e003461.**
  This study also highlights the high prevalence of D2T-axSpA, affecting nearly 20% of patients exposed to bDMARDs. It underscores the association of D2T-axSpA with hypertension, depression, and peripheral arthritis, offering a broad perspective on the disease burden.

## Data Availability

No datasets were generated or analysed during the current study.
